# Views on social media and its linkage to longitudinal data from two generations of a UK cohort study

**DOI:** 10.12688/wellcomeopenres.15755.2

**Published:** 2020-08-12

**Authors:** Nina H. Di Cara, Andy Boyd, Alastair R. Tanner, Tarek Al Baghal, Lisa Calderwood, Luke S. Sloan, Oliver S. P. Davis, Claire M. A. Haworth

**Affiliations:** 1 MRC Integrative Epidemiology Unit, University of Bristol, Bristol, UNITED KINGDOM, BS8 2BN, UK; 2 Department of Population Health Sciences, Bristol Medical School, University of Bristol, Bristol, BS8 2BN, UK; 3 Institute for Social and Economic Research, University of Essex, Colchester, Essex, CO4 3SQ, UK; 4 Centre for Longitudinal Studies, University College London, London, WC1H 0NU, UK; 5 School of Social Sciences, Cardiff University, Cardiff, CF10 3AT, UK; 6 The Alan Turing Institute, British Library, London, NW1 2DB, UK; 7 School of Psychological Science, University of Bristol, Bristol, BS8 1TU, UK

**Keywords:** Social media, data linkage, cohort study, social licence, acceptability, ALSPAC

## Abstract

**Background:** Cohort studies gather huge volumes of information about a range of phenotypes but new sources of information such as social media data are yet to be integrated. Participant’s long-term engagement with cohort studies, as well as the potential for their social media data to be linked to other longitudinal data, may give participants a unique perspective on the acceptability of this growing research area.

**Methods:** Two focus groups explored participant views towards the acceptability and best practice for the collection of social media data for research purposes. Participants were drawn from the Avon Longitudinal Study of Parents and Children cohort; individuals from the index cohort of young people (N=9) and from the parent generation (N=5) took part in two separate 90-minute focus groups. The discussions were audio recorded and subjected to qualitative analysis.

**Results**: Participants were generally supportive of the collection of social media data to facilitate health and social research. They felt that their trust in the cohort study would encourage them to do so. Concern was expressed about the collection of data from friends or connections who had not consented. In terms of best practice for collecting the data, participants generally preferred the use of anonymous data derived from social media to be shared with researchers.

**Conclusion**: Cohort studies have trusting relationships with their participants; for this relationship to extend to linking their social media data with longitudinal information, procedural safeguards are needed. Participants understand the goals and potential of research integrating social media data into cohort studies, but further research is required on the acquisition of their friend’s data. The views gathered from participants provide important guidance for future work seeking to integrate social media in cohort studies.

## Introduction

The analysis of data collected from social media is a rich and growing area of current research in a wide variety of fields. Social media data has been used to track the spread of disease ^1^, predict the results of key elections ^2^ and gauge public reaction to events ^3^. Not only are these data widely accessible but they provide a wealth of rich information on views, feelings and interests. Whilst these data are highly valuable in health and social research there are few reliable data sources that can link social media data to factual information about users’ lives, with most applications so far focussed on identifying broader trends using ‘big data’ methodologies. Without these so-called ‘ground-truth’ (empirical, rather than inferred) data, it is not possible to adequately validate social media sentiment analysis methods, or to infer the relevance of the patterns observed to the general population ^4^. At present, longitudinal population studies (LPS) remain an untapped resource in terms of obtaining this empirical information. Conducting social media data linkage in this way also has the potential reciprocal benefit of adding significant value to the data already available in the LPS. Those participating in LPS are already familiar with the process of collection and use of sensitive data, have evidenced commitment to providing their personal data for the advancement of science, with these data being readily accessible to researchers. As highlighted by Wellcome ^5^, and the Medical Research Council (MRC) ^6^, a key future direction for such studies is to conduct more data linkage. Data linkage within existing and prospective datasets has the potential to reduce the burden on participants and maximise the benefit of research data collected ^5^, whilst using new types of data that allow for remote data capture, such as social media, is hypothesised by the MRC as a method that could address a lack of engagement and offer cost-effective modes of data collection ^6^. We note that the definition of the term ‘social media’ is left to be explored and defined by the participants within this study.

Whilst such data sources present exciting possibilities, organisations and those working in the emerging population data science field are conscious of the need to understand public views and expectations around the novel use of such data in research ^7, 8^, and that a process of public/participant dialogue is needed to ensure new activities do not undermine trust in the study and can be seen to provide public benefits ^9^. Within the UK the failure of the care.data program is cited as a reminder that even where data science initiatives are legal and technically feasible they can still fail if they lack the ‘social licence’ needed for public and key stakeholder support ^10^. Existing research in the field of record linkage, outside of social media linkage, has found that there is a general acceptance of this work from the public ^11– 13^, even when conducted without consent if data is appropriately anonymised ^13^, but that these decisions are ultimately complex and conditional on the situation ^9, 11– 14^. Therefore, it is essential that any novel data linkage activity, or a novel use of existing data, is informed by exploring participant views towards its acceptability, as well as researchers exploring the participants understanding of the data and how it will be used. In this manner participants can inform study’s efforts to reach a consensus on the best practices for collecting these potentially sensitive data and sharing these with researchers in a secure and ethical way that protects participant anonymity.

The use of social media data for research has also had its own ethical challenges concerning privacy and informed consent ^15, 16^, as well as difficulty defining what mediums are included in the definition of social media at all ^17^. A systematic review by Golder *et al*. ^18^ in 2017 found that social media users and researchers tended to be conflicted about whether informed consent was necessary for data collected from public social media sites and, similarly to data linkage issues, this debate tended to rest on the nature of the content, which source the data came from and how the data would be used ^14, 19– 21^. Subsequent ethical guidelines developed for the field have placed special consideration on the reporting of social media research to ensure user’s privacy ^22, 23^, and reflects participant’s views that increased sensitivity and personal identifiability of the subject matter should increase the level of anonymity with which it is reported ^15, 19, 24^. Previously, research participants have found that photos are more personal than text data ^19, 25^, however the level of trust in the study or the researchers conducting it may also influence their decision of whether or not to share ^10– 12^. There is evidence to suggest that there are a body of users who expect their data to be collected as ‘necessary evil’ of day-to-day social media use; these users tend to see information privacy as the responsibility of the individual rather than the company holding the data ^15^. There may also be an age related aspect to participant’s willingness to share their social media data, with younger people more likely to agree ^26, 27^.

Collecting social media data from LPS therefore appears to be promising, as participants will always have given explicit consent, are likely to have a good awareness of how their data is kept safe, and have trust in the study to use and report their data responsibly. This may mean their agreement to share their data and link it to existing data is more likely. However, it is particularly important in these studies to maintain the trust that has been built with participants by co-creating an understanding of what is acceptable with regards to their information, especially since LPS participants may have specific concerns about the linking of their social media activities to the large volumes of diverse sets of data already held about them by the study. In addition, the series of high-profile online data scandals, the introduction of the new General Data Protection Regulation (GDPR) and the significant concern about the manipulative political use of social media data in what has been called the “Cambridge Analytica scandal” may have had the potential to influence participant’s views about what they consider to be acceptable in terms of data collection, linkage and reporting on their social media data ^28^. As such, this study into participant’s views aims to ensure our knowledge of what is considered acceptable practice for social media data linkage remains current in the evolving landscape of online privacy, and to ensure that we consider the specific views of participants in LPS.

In this study we report on participants’ views on social media data linkage in an on-going birth cohort study, the Avon Longitudinal Study of Parents and Children (ALSPAC), also known as ‘Children of the Nineties ^29– 31^. Focus groups were held separately with participants from the index offspring cohort, who are now in their late-twenties, and with the parent cohort, and included semi-structured discussions on their views on, firstly, how they would define social media and what they use it for, and then their opinions on social media research and data linkage. Due to the ambiguous nature of social media we made it a priority to first understand how participant’s view it as a medium and how they report interacting with it, prior to trying to interpret their views around their data privacy.

## Methods

### Sample and Recruitment

ALSPAC is a transgenerational prospective observational study which recruited pregnant women living in Avon, UK; those with expected dates of delivery between the 1 ^st^ April 1991 and the 31 ^st^ December 1992 were invited to take part ^29– 31^. The initial number of pregnancies enrolled was 14,451 and of these pregnancies 13,988 children were alive at one year of age. This was supplemented when the index children reached approximately age seven where eligible cases who had not joined originally were invited to the study, resulting in a total of 14,901 children alive at one year of age for which there is data from age seven. Since joining the study both parents and index children have been routinely assessed on a number of environmental and psychological measures, provided biological samples and their genetic data. The wide variety of longitudinal data from both generations has provided valuable opportunities for a breadth of research into health and social outcomes for children and young people, as related to genetic, environmental and social factors. Please note that the study website contains details of all the data that is available through a fully searchable data dictionary and variable search tool ( http://www.bristol.ac.uk/alspac/researchers/our-data/).

For the purposes of this study, we recruited two separate participant groups from the available sample to take part; the first contained the index children themselves, and the second group contained participants from the parent cohort. A random sample of participants in the study who lived in the Bristol area were invited to take part, to allow easy access to the study location. Inclusion criteria were that participants had a social media account, and spaces were filled on a first-come-first-served basis. The index child group was made up of nine participants aged 26 to 28, with four males and five females. The parent group was made up of five participants aged 53 to 65, with one male and four females. All participants were reimbursed for their travel expenses and offered a £10 shopping voucher for taking part.

### Ethics

Ethical approval for this study was provided by the ALSPAC Ethics and Law Committee and the Local Research Ethics Committee, at the University of Bristol. All participants gave their written consent for participation and audio recordings. Fair processing information describing the study was provided in a postal invitation pack. Participant’s consent was obtained on arrival at the focus group.

### Data collection

Data were collected using focus groups, defined as “semi-structured discussions of 4–12 people that aim to explore a specific set of issues” ^32^. Focus groups were seen as the most appropriate data collection method, as opposed to surveys or one-to-one interviews, due to the ability to resolve and discuss conflicting views and information through group interaction, clarify individual and shared perspectives, as well as directly explore the relative emphasis on certain topics in order to understand their subjective importance ^32, 33^.

Two focus groups, one for each generational group, took place consecutively at the ALSPAC offices on the morning of Saturday 22nd September 2018. Each focus group lasted 90 minutes and were led by the Principal Investigators of the study Dr Haworth [CH] and Dr Davis [OD], who were assisted by three members of their research team [AT (PhD), JA (MSc), ND (MSc)] and one member of ALSPAC study staff. None of the facilitators had previous relationships with the participants. Both Principal Investigators have previous experience in conducting focus groups and provided guidance to the assisting members of the research team. There were three female [CH, JA, ND], and two male [OD, AT] facilitators present. The participants were made aware that those present were interested in the potential of social media to improve health and wellbeing, and were later introduced to previous research into expressions of happiness and anxiety on Twitter during the presentation given by Dr Davis in the middle of the group. Beyond this they were not made aware of the facilitators’ specific research interests, which we are reporting in line with the CORE-Q criteria for qualitative research ^32^. Throughout the focus groups the facilitators used a ‘funnel’ style approach to questioning by starting with general questions about individuals’ views on what they believed social media to be and how often they used it and then becoming more specific as topics of interest were narrowed down.

The focus groups were structured into the following three parts.

**Part 1: Personal views on social media in the UK today**

After basic introductions, we introduced a discussion on what the participants believed social media to be, how they tended to use social media, and what they used them for. Participants were subsequently asked to classify themselves as high, medium or low social media users based on the definitions proposed by NatCen ^19^ as seen in Table 1, and their own understanding of what social media are.

**Table 1.** Definitions of levels of social media use as given by NatCen ^19^. The definitions given were used in both focus groups to identify the range of social media use represented by the participant group.

Level of Use	Description
High	Those who use social media several times a day
Medium	Those using social media from twice a week up to once a day
Low	Those who do not use social media or use them once a week or less

**Part 2: Presentation on applications of social media data**

The participants were given a presentation (Supplementary Material 1, *Extended data*) ^34^ on the applications of social media data in health and social research which included examples of population level disease symptom tracking, and sentiment analysis of Twitter data, as well as then introducing the spectrum of identifiability of data, using resources from ‘Understanding Patient Data’ ( https://understandingpatientdata.org.uk/what-does-anonymised-mean), which clarifies the difference between raw, de-personalised and anonymous data. These three terms were understood in this study as:

*Raw*: Information in its original format, for instance a status update, with no attempts to remove identifiable information.

*De-personalised*: Information which has had identifiable features removed, but still contains individual information.

*Anonymised:* Information that has been processed, for instance into a numeric score or aggregated, so that there is no recognisable association between an individual and the piece of information.

**Part 3: Views on using social media data for research**

**Figure 1.**
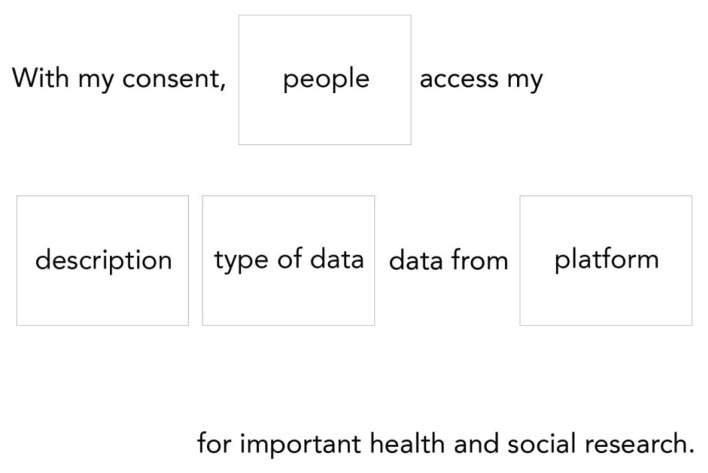
The template that participants filled with options from Table 2 to provide discussion points. This template was used by participants, in conjunction with the options from Table 2, to discuss their views around a wide range of different data access scenarios. This allowed the research team to unpick which types of variation in the scenario might make it more or less acceptable to the participant group.

**Figure 2.**
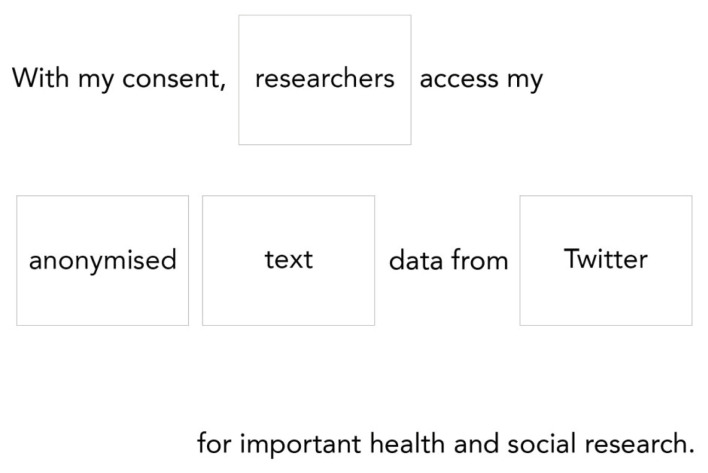
An example of a completed template from the situational exercise. This provides an example of how the templates were used during the focus groups to explore a particular situation in which their data might be accessed. Variation of the item in any one of the boxes could be changed to explore how this might alter the participant’s opinion on the scenario.

Following the presentation, we provided each participant with a template as presented in Figure 1, with ‘blank’ spaces which could be filled with cards labelled as described in Table 2 to form a possible research scenario. This exercise could produce up to 108 unique scenarios for discussion which were designed to explore participants’ views around linkage of different types of data, and how they would expect these data to be shared and presented to different types of researchers. To illustrate, an example of a completed template is given in Figure 2. Participants chose the cards at random from a pack to minimise the bias in the selection of options, then discussed the scenarios in small groups of 4–5, with a facilitator moderating each group.

**Table 2.** The options presented to participants to fill each ‘blank’ in the statements in Figure 1. One option from each column was randomly selected by participants to complete an activity which explored their views on possible scenarios in which different types social media data might be accessed.

People	Description	Type of data	Platform
‘Children of the Nineties’ staff Researchers Computers	Raw Depersonalised Anonymised	Friends Network Likes Text Images Location	Facebook Instagram Twitter

### Analysis

Immediately following the focus group, a short debrief was held between the members of the research team to collate their experiences and any trends they had noticed. An analysis of themes was then later completed from the audio recordings by one researcher [ND]. The analysis procedure was first, familiarisation with the data through listening to the recordings of the focus groups and transcribing them, then identifying material that was not neutral (neutral material included comments from facilitators, or conversations which were not relevant) and finally systematically coding the relevant material and organising it into themes. Due to the semi-directed nature of the focus group, and relatively small volume of data, the data were first coded deductively ^35^ into the existing structure given by Part 1 and Part 3 of the focus groups above, rather than inductively deriving broad themes. Within the sub-theme ‘What are social media used for?’, participant responses were inductively coded and summarised into themes. As such the results are presented as narrative summaries of participants of the two parts, with any identified sub-themes as relevant.

The software package NVivo 12 was used in the analysis stage in order to code the participants’ comments digitally and allow for comments on particular areas to be viewed collectively. Participants were not invited to check the results following their participation in the focus group, but we reviewed all quotes to ensure that participants could not be identified.

## Results

The following results are a narrative description of the outcome of our analyses, arranged by the two discussion sections of the focus groups, and by each main topic within these sections. As such, the first section describes the participants’ general views on social media and how they use it. The second section then summarises their views on the use of social media in research with respect to the different variables presented to them in the template exercise, given in Table 1. Quotes have been provided where relevant to further illustrate the discussion.

### Personal views on social media in the UK today

**What ‘counts’ as social media, and who is using them?**

The groups were first asked to discuss what social media was. This prompted both examples of platforms, and descriptions of what made certain platforms ‘count’ as social media. In both groups there was an agreement that defining an application as a type of ‘social media’ was dependent on whether it was possible to share content **and** interact with others. For instance, the young people agreed that WhatsApp was a type of social media only due to recent updates that allowed users to share ‘daily stories’ and status updates, rather than solely message specific individuals.

*“I would just say anything where anybody could join in a, sort-of, general discussion.”*
“ *Anything where you socialise through media… yeah” – Parents*

Facebook was the most widely used and discussed form of social media in both the young people and parent groups, shortly followed by Instagram and Twitter. This is consistent with Facebook being the most common site for adults to have a social media profile on 36. The parent group shared a mutual agreement that they had all had Facebook for the longest time, and preferred it as it was “easier” and more “familiar” than alternative platforms; some participants in this group stated they had had Facebook for over 10 years. In both groups many of the participants stated they were WhatsApp users too. Proportionately more of the young people described actively engaging with Instagram than the parents, and whilst several people across the groups stated they had Twitter profiles, the majority of those said they looked at what other people posted rather than creating content themselves. A sub-section of the younger group noted that they did not regularly post to Facebook, and agreed between them that Facebook was used less by young people than it previously was; this observation is consistent with reported data on the changing demographics of Facebook users ^37^. A number of the young people stated that they use Snapchat, and whilst the parent group were aware of Snapchat none of them were users of this platform.

*“Facebook mainly. That’s probably the only one I use… and Whatsapp.” – Parent*
“ *Facebook seems like a platform for older people now.*”
“ *Exactly, my mum uses Facebook more than I do.” – Young People*

Some of the parent generation identified fitness tracking apps such as FitBit and Strava as their more commonly used social media and noted that setting challenges for other users and participating in group competitions were social elements they enjoyed. Several of the younger generation also used fitness apps.

***What are social media used for?*** In the parent cohort all five participants identified themselves as ‘high’ social media users. In the younger cohort seven out of the nine were also ‘high’ users, with the remaining two identifying as ‘low’ users. These categories of use followed the definitions given by NatCen ^19^ in their 2014 report where they had equal numbers of participants in each user group, but the proportion of ‘high’ users was much larger in this study. This may represent sampling bias in the present study, and the inclusion criteria of being a social media user may have dissuaded ‘low’ or ‘medium’ users from taking part. However, there was still a wide range of frequency of use within the ‘high’ user group, from checking Facebook once a day on a laptop to using it on waking and then multiple times a day on a portable device. This may suggest that the definition of ‘high’ use is no longer representative given increases in social media use and prevalence over the past five years ^36, 38^.

When asked to explain what they ‘do’ on social media participants discussed a wide range of activities, which have been individually set out and summarised below.

**Interacting with friends and family**

The main use of social media reported was to keep in touch with friends and family, especially those who were not easily accessible to meet with face-to-face. This was consistent across both groups.

*“I use Facebook for like similar to you just like keeping in touch with family and friends that I wouldn’t necessarily see.” – Parent*

Several participants noted the ability to “keep in touch” with others online but the means of communication was not always clear. Some participants clarified that they would have conversations on a private messaging feature of the platform, such as Facebook Messenger, whereas others described it through interactions on content posted to their profile.

*“I don’t speak to [my friends and family] directly but they like the things I post.” – Young Person*

**Posting content**

Several participants gave examples of posting content to social media. These examples included pictures of evenings out, asking questions to their online network, pet updates, and exercise records on apps such as FitBit or Strava. As well as personal content a handful of participants also mentioned sharing articles and news through their profiles.

**Competitive activities**

There were two types of competitive activities described by the participants. One was playing games through social media, and the other was taking part in competitions with others on fitness apps. The discussion around fitness competitions was specific to the parent group.

*“So when you say about health and fitness there are some amazing apps that you can socialise with other people. And I think they encourage you, or you compete.” – Parent on fitness apps*

Games were discussed in both age groups, but most of the parents described engaging with online games which operate through social media, usually Facebook, compared to a minority of the young people. The games included Scrabble, Candy Crush and FarmVille, with some using these as a way of engaging with friends and relatives on a regular basis.

*“I actually use Scrabble [on Facebook] to keep an old lady company every night” - Parent*

**Events**

Using Facebook as a platform for event planning and organisation was raised between the young people, but not among the parent group. The types of events included those within their immediate social network as well as entertainment advertised through Facebook.

*“I use Facebook to know what’s going on in terms of events, the biggest thing I would use it for is going to a party. It’s so useful for ‘there’s a band playing’ or your friend is having a party. I think for me that’s Facebook’s biggest use.” – Young Person*

**Passive or observational use**

Whilst the participants used social media to stay in touch with others, many of the behaviours described involved ‘scrolling’ through Facebook or Twitter as a consumer of other people’s news.

*“I have Facebook but I can’t remember the last time I posted anything on there” – Young Person*
“ *I’ve got a Twitter account but all I do is look at stuff, I never actually put anything on Twitter” - Parent*

This passive social media use could be reflective of reported increases in social media being used as a vehicle for viewing content such as videos and news, along with decreases in users creating content ^39^ which may be to avoid negative feedback on their own user-generated content. The participants described using social media to stay up to date with news articles and celebrities as well as news in their social networks. In parallel with this the young people discussed their perceived need to be discerning about where they got their news from, and which sites they regarded as more trustworthy; there was a disagreement between participants about whether Facebook or Twitter was the more trustworthy news-source and views appeared to be based on preference rather than experience or evidence.

*“I would have more faith if something was trending on Twitter than a load of random news articles on Facebook.” – Young Person*

In both groups there were references to using social media to find information about or posts from other people. This is colloquially known as ‘Facebook stalking’ ^40^. The parent group were open about using this feature to see what their children were doing, and also to research their children’s friends and romantic partners. The young people discussed the impact of this phenomenon on dating and how image management on social media may result in gaining an inaccurate impression of what a potential date may be like in real life.

”I stalk my children!”
“Yes, I do stalk my children’s boyfriends” – Parents on ‘Facebook Stalking’
“ *Dating apps are part of social media. Some people will research the person they’re going to meet.” – Young Person*

***Opinions on social media in general.*** There were a wide variety of opinions about social media platforms in general. Conversations about concerns were far more prevalent in both groups than discussion of the positive aspects of social media, and both groups were critical about the potential implications of social media. In the vast majority of the participants’ conversations the concerns were speculative, in some cases directly observed and there was only one example of someone being directly impacted by cyber-bullying. We note that not all participants may have been happy to speak about this in the group setting.

In the parent group the main concerns were about the behaviour of others online. One example given was the use of Snapchat by young people to bully others by reaching a large number of people very quickly, and other examples were given around behaviours of others their own age posting derogatory comments on other people’s content. Those present expressed their disdain for this behaviour, and hypothesised that the anonymity of social media allowed others to act in this way.

*“Sometimes I look at the comments people in my age range put up, and I think ‘wow, would you say that out loud in a room full of people’?” – Parent*

The parent group also expressed concern about the immediacy of social media, and the “pressure” to respond instantly to messages and communication online. There was a feeling that this would be difficult for younger people to manage, though was not a concern raised by the younger group.

The younger group’s concerns were centred around their inherent distrust in their data security on social networking platforms, as well as the way that people’s lives may be curated on social media with a bias towards positive events. This was also noted by the parent generation as a risk for young people’s mental health when using social media.

“ *If you got engaged and put it on Facebook that’s immediately like 500 likes, it’s blown out. [If you put] ‘I’ve had a really rough day and my dog is sick’, maybe 1 like. We’re so busy chasing the happiness that the duality is never there.” – Young Person*

Amongst the younger generation there was a general consensus that for those who were high users of social media their ‘offline’ and ‘online’ worlds were inherently linked, from the events they choose to attend in the ‘offline’ world to the conversations they had with friends. However, there was debate amongst the young people on whether the representations they made of themselves online was a genuine or biased reflection of their true selves. Some openly acknowledged that they preferred their online persona, whereas others did not feel there was any difference between their online and offline selves. One positive that the young people noted was the ability to curate their online appearance for employment sites such as LinkedIn when desired.

***Summary.*** The majority of participants were ‘high’ social media users ^19^ and used similar social networking sites across both groups, such as Facebook and Twitter, though with more younger people using Instagram and Snapchat. The main reason participants used social media was to keep in touch with friends and family, but this was being performed in different ways including playing games, posting content and looking up other people on social media sites. Participants also described getting news and information from Facebook and Twitter. Both groups expressed concerns about the role social media plays in their lives and the lives of others, with anecdotal examples of risks of social media use to personal wellbeing, information security and obtaining misleading information.

The discussions highlighted that there are myriad types of data possible to link, and that which of these data we choose to link may impact participant’s views on data linkage. In the next section we explore participant views on specific types of social media data linkage.

### Views on using social media data for research

After being given a presentation on the possible uses of social media data, participants took part in a novel exercise where they discussed different possible scenarios for data collection, with the variables as given in Table 2. The participants engaged well in this exercise and distributing the possible options for each variable amongst the group encouraged a rich, collaborative discussion around what would be deemed acceptable. The results of the analysis below are grouped by each potential variable that participants were asked to consider.

***Who has access to the data.*** Participants discussed how they would feel about different people accessing their data, with the options being ‘Researchers’, ‘Children of the Nineties staff’ or ‘Computers’. Participants understood ‘Researchers’ to be those not necessarily affiliated with the study who may apply to use their data for health and social research, with the study staff being the administrative staff who facilitate the study. No participant had any concerns about the data stored about them being accessed by the staff or researchers associated with the cohort study, with several participants voicing their trust in the study and its protection and safe use of their data. As such, they trusted that their data would not be sold on or used for purposes other than health and social research, which in other contexts was a widely expressed concern in both groups, but more prominently in the young people.

*“’Children of the Nineties’ is fine because I know you’re not going to sell it.” – Young Person*
“ *I would trust emphatically the ‘Children of the Nineties’.” - Parent*

Out of the three possible options most discussion was had over the use of computers (i.e. automated harvesting of information) in accessing participant data, with evident confusion over what that meant in practical sense for both participant groups, and easy misinterpretation of what it would be possible for computers to achieve with their data. For instance, most participants needed clarification on exactly how a computer would be able to access their information, and in one instance there was a concern from participants in the parent cohort that a computer could access and manipulate layers of private data whilst mining social media platforms.

*“Once it gets into your systems it can get everything else it wants out of it. Once you’ve put something into Google it can find every email you’ve sent.” - Parent*

The facilitators provided clarification that the cohort research staff would not be able to access private user information held by the social media platform, only the data that would be available to anyone accessing Twitter information. Facilitators also clarified the difference between a malicious virus on a computer and authorised means of accessing online data through an application programming interface. This was generally understood, but some participants were still uneasy about the use of computers. Ultimately, when asked at the end of the sessions if they would be happy for computers hosted by the cohort to collect their Twitter text data all participants agreed they would consent to this.

***Level of data anonymity.*** A difference in attitude between the groups characterised the concerns around the level of data anonymity consented to in each scenario. The parent cohort mentioned on several occasions that they would not post anything on social media that they were not happy for the public to see, and that they regarded it as a public platform. This appeared to lend a relaxed view to all those attending the focus group towards sharing their data in any form, once it was clear that the data would not be sold on and that only the information they consented to sharing would be collected. However, some members of the group said that they would feel more comfortable for computers to have only their depersonalised data due to concerns about what computers could theoretically do with their information; this was related to a misunderstanding about what computers could do as described above.

The younger cohort appeared more discerning about the level of data they were willing to provide, with several noting that they felt “safer” or “happier” when raw data was not being used.

*“I suppose when things become anonymized it all seems a lot more fine. If it’s being reduced to numbers and data points I would be much more likely to give my consent.” – Young Person*

It was necessary to clarify for the participants that their private messaging data would not be collected, and that computers would only collect the data they had agreed to. Once this had been explained all participants agreed they would be willing to provide their personal text and ‘like’ data in any form. Photo data created more discussion and the younger participants generally felt they would require more anonymity with photo and location data. Anonymity of photo data in this sense referred to distilling a photo down to numeric data, such as the hues and colours used in an image, or to a description of the image.

***What types of data are acceptable to collect.*** The type of data collected generated the most discussion in both groups. In principle the vast majority of the participants agreed that they would provide all forms of data ( Table 2), apart from friends’ data, given that it would be collected and distributed by a study that they trusted to store and use their data well. Here, ‘friend’s data’ refers to data produced by a friend or connection, rather than data produced by the user about their friends such as a list of connections, which would be considered ‘network data’.

In the younger cohort there was general agreement that text and ‘like’ data could be collected. Photo data created more discussion, with a feeling among some that it was more personal than their text data, and some feeling that if it was anonymised in the same way then it was no different to text data.

*“There’s something intricately linked between your privacy and yourself. A picture of yourself is much more private and identifiable than anything I would write down. So definitely I think I would feel uncomfortable” – Young Person*
“ *If the photo is just being broken down into a code then it’s the same as text. What’s being taken and the final product is the same thing.” – Young Person*

Other reservations about photo data included a distrust in the ability to reliably code the features of a photo with the technology currently available, as well as concerns about involving friends who are in the photos without their consent.

In the parent cohort, the same concerns about photos were not expressed, with all agreeing that they only post photos that they would be happy for anyone to see. A similar view was demonstrated by some members of the younger focus group too, given an awareness of how their social media data might be seen by others.

*“The only photos I put up on Facebook are ones I’m happy for the whole world to see” - Parent*
“ *I’m conscious that any job I go to is going to investigate it [social media profile] themselves so I don’t think anything is very private. I’m always very conscious about what I post.” – Young Person*

The younger cohort had some discussion about location data, but ultimately did not express concern since the majority of participants did not regularly ‘check-in’ on social media or report their location. Those that did were not concerned about the study accessing this information about them and did not feel it was any more personal than the other data the study held on them.

The area of data collection that caused the most concern was data related to their friends accessed through their account. For many of the participants this did not vary across platforms, even if all the data collected would be public anyway, for instance on Twitter. Both sets of participants felt uncomfortable at the idea of giving consent for others to access these data when their friends had not agreed, as well as concerns in the younger generation about ownership of data and what would happen if those data had been collected by the study but the original content was later deleted by the user.

*“I object to it strongly... my friends haven’t agreed to that.” – Parent*
“ *Twitter is public, but only for the time you leave it up. If you give permission for someone to store and access your data you’re giving them permission to have it for as long as they want it.” – Young Person on giving consent for collection of friends’ data*

When a sub-group of the young people were explicitly asked whether they would consent to the positivity or negativity of their friend’s posts being anonymised and scored in order to gauge whether this impacted on their own posts they agreed to this, which was a contradiction to the same group previously stating they would not consent to any of their friend’s data being accessed. Their agreement was more readily given for Twitter than for Facebook. Some participants actively voiced their dilemma, that they knew these data would be “used for good” but that they felt morally conflicted about actively allowing it. Other participants reasoned that they would probably give their consent, and that the focus group environment was encouraging them to think more deeply about it than they usually would. Collectively the sub-group of young people came to a decision that they would “probably” allow for these data to be collected, on the basis that it would be used for “important health and social research”.

*“We’re always sharing our data with loads of people all the time who are using our data for advertising and selling it on. At least with this we would have given our consent and knew it was for a good cause.” – Young Person*

***Collecting data from different social media platforms.*** Across both cohort groups there was a consensus that though they had preferred platforms they would ultimately consent to sharing their information from any platform once they understood how the data would be protected and what it would be used for. This agreement was usually given with reference to their trust in the study that their data would be safe.

*“Anything I was sharing on my social media, including location, I’d be happy to share with ‘Children of the Nineties’” – Young Person*

When considering friends’ data, this did not differ across platforms, and participants were mostly consistent that they would not provide consent for this. This was with the exception of the sub-group who expressed more willingness to consent to their friend’s data being collected from Twitter, as a public platform, than Facebook. This subgroup ultimately stated that they would consent to this data being collected from both platforms if it was being used for ‘good’, though their initial reaction was that this would not be acceptable to them.

“ *I wouldn’t be happy if someone consented on my behalf. And that’s the same on every platform. It’s not my place to consent on someone’s behalf.” – Young Person*

***Data linkage.*** Data linkage refers to joining together previously unassociated information about an individual in order to build a comprehensive collection of data about them from different sources, for instance attaching health data to educational outcomes. In this study this would involve adding relevant social media data to each individual’s cohort study data profile for use by researchers (which can include information from health and other official records). When asked whether this would be acceptable, many of the participants had already assumed that their data would be linked if it were collected as part of the study, and were agreeable to this happening. In fact, all the participants agreed that this would be the best way to get the value from their data and had ideas about which research questions might be answered by doing so. This view was consistent across all participants in both groups.

*“I think it’s important. Because to get the fullest roundest picture you need to do that anyway don’t you.” – Parent on linking their existing data*

***Views on suggested research methodology.*** The final part of the focus group was suggesting a possible research methodology to participants, to see if they would be inclined to agree to it. The methodology was threefold, and in each case was presented as being with participants’ consent for use in important health and social research:

- “Computers access my raw text data from Twitter”
- “Children of the Nineties staff access my raw text data from Twitter”
- “Researchers access my anonymised text data from Twitter.”

Participants unanimously endorsed this arrangement, understanding that study staff could hypothetically access their personal data but that they would not routinely need to do so. Participants reasoned that this was equivalent to the requirement for study staff to access any of their other sensitive data held by the study, such as health evaluations.

***Summary.*** On the whole participants agreed they would be happy to share their text, ‘like’, location and network data with researchers from the study in any form, though it was deemed easier to accept the further the data were anonymised. Photo data were a slightly more sensitive data type, with the majority agreeing they would share this in its raw format, but a minority considering photo data too identifiable to share unless anonymised. Participants frequently cited their trust in the ALSPAC study, and subsequently their trust in anyone who was given the data, though there were reservations and confusion for a small number of the older generation participants on the role of computers in the data collection process due to misunderstandings about what computers could do. As participants were agreeable to sharing most of their data, they did not have reservations about which platform this was done through; however, the platform had some influence when considering whether to share information about their friends. For instance, some participants felt it would be more acceptable to collect information posted publicly to Twitter than to Facebook private profiles. The majority of participants were not agreeable to allowing collection of their friends’ data, and those who did agree only did so when given a clear scenario describing the type of research question that this would be useful in answering and the anonymisation approaches that may be used.

When considering the differences between generations, the groups’ levels of technological insight had a varying effect on their opinions and thresholds for agreement. For instance, in the older group the use of computers in the research generated unease due to the perceived likelihood of them distributing malicious viruses or leaking personal data. The older generation agreed they would be comfortable for the study to collect any data, apart from friends’ data, because they had nothing to hide. In the younger group the privacy of the actual content was not widely considered, however the issue of information security and desire to protect their private information was more apparent. Along with their knowledge of this area came a sense of inevitability of how often loss of privacy already happens, and some reference to incidents such as the Cambridge Analytica scandal. The participants’ willingness to share their data was then because they were aware that their data were already being used for the benefit of private companies and they would rather it was being used ‘for good’ as well.

All the participants also showed good insight into how the data could be useful to the cohort study, with several participants offering suggestions of how their social media data could contribute to health and social research and other platforms (such as exercise or dating apps) that the study should consider researching.

## Discussion

The question of how participants in cohort studies feel about sharing their social media data has been largely unexplored, but the majority of the findings from the present study are consistent with those from previous focus groups on users’ views of data linkage and social media research ^19, 41^. Ultimately all the participants agreed that they would consent, if asked, to the study collecting their social media data in a scenario where computers (managed by study staff) accessed the raw data, and converted this into anonymised numbers which were then distributed to researchers. They would also consent to these data being linked to their existing data in the study. The most acceptable data types to collect were text, ‘likes’, location and network data, with images being slightly less acceptable to some, and friends’ data being particularly contentious, with only a minority agreeing. When discussing the use of friends’ data, participants’ views changed depending on how the question was phrased. When first asked if they would share their friends’ data, participants were firm that they would not. However, some agreed when presented with a specific scenario. The participants noted their own difference in opinions, and despite discussion around this there was no overall resolution of opinions for any given situation.

The findings on the participants’ general views on social media showed similar themes to those noted by O’Reilly and colleagues ^41^ in their focus groups with adolescents, where participants held a view that social media is bad for mental health, that it was a platform for bullying and some reference to the ‘addictive’ nature of social media. Certainly, most of the discussion about social media was about its negative attributes, with words such as ‘dangerous’ and ‘unsafe’ used. The belief that social media can be detrimental extended to those participants who said that they had not directly experienced negative consequences. However, the participants’ acknowledgement of the negative side of social media was held alongside specific examples of the benefits such as keeping in touch with friends or providing company to lonely older people. This illustrates the participants’ awareness of the advantages and disadvantages of social media, and represents a considered decision to continue engaging with it.

As well as a generally negative view of the impact of social media, there was distrust of online data security amongst both generational groups. In the older generation this presented as concerns about the use of computers in the research, and in the younger cohort presented as increased awareness of their digital privacy on social media sites and the inevitability of the exploitation of their data, which appeared to make them more discerning than the parent group on what they would agree to share. This could be seen as consistent with the younger group having grown up with technology available to them and having a different awareness of how it operates than their parents do. However, this view of younger generations as ‘digital natives’ ^42^ can be misleading; whilst age is associated with someone’s likelihood to be immersed in technology, it is not the only relevant factor ^43^. As such the generation differences we observed may not solely be attributed to the participant’s age. Interestingly, this contradicts findings by Wellcome ^25^ on the public’s views of general data linkage, where younger people were more likely to agree to share their data, and older generations were less likely. A broader sample of participants would be helpful in order to thoroughly investigate the nuance in the reasoning of both groups, and how it relates to the types of data being shared.

Despite having some privacy concerns about their social media data, situating this type of data against the level of privacy of other data held on them in the cohort study, such as health assessments and genome-wide genetic data, allowed participants to make reasoned and informed judgements about what they would consent to. However, within ‘social media data’ there were layers of types of data which held different levels of sensitivity to participants and, similarly to reports by both NatCen ^19^ and Wellcome ^25^ on users views, photo data was slightly more sensitive than other types of data. NatCen’s report found that researcher affiliation had an impact on whether a participant would consent to a scenario, and we saw this influence with the ALSPAC participants who were openly confident in the study and its intentions and told us that this gave them confidence in sharing data with the study. We hypothesise that a study which is not using an LPS sample may find more resistance to the disclosure of those data considered more sensitive. Similarly, while the NatCen participants ^19^ had reservations around the efficacy of social media research the participants in the present study displayed accurate insight into how their data may be useful to researchers and why it was important to gain their views, which could be attributed to their long-term participation in the study.

A common theme throughout both focus groups was reference to their trust in the study, and their belief that their data would be used to benefit others, which supports the use of LPS as a valuable source of ‘ground-truth’ data due to participants’ existing investment in participating in research and the depth of data already available on the cohort. The differences in concerns between generations suggests a need for informed consent to be obtained in a thoughtful and well explained way which meets the needs of all age groups, particularly those who are not ‘digital natives’ ^16, 42^.

The variety of opinion around the use of friends’ data which were found in this study warrant further exploration, particularly given the current digital privacy environment, and the apparent lack of concern over the use of ‘network’ data such as lists of friends. The difference in opinion depending on how the question was phrased may suggest that only specific, controlled scenarios are acceptable to participants and understanding the thresholds of this decision making is important in considering the ethical implications of this type of work. Similarly to other work on non-consensual data linkage ^13^, the differences in stance may also be a reflection of the complexity of the decision and the ethical dilemma it presents to participants.

There were limitations to this study, particularly around the sample of both ALSPAC and of the focus groups in particular. Although Bristol was at the time of recruitment representative of UK cities, there is estimated to be a shortfall in the recruitment of less-affluent families, and mothers from ethnic minority backgrounds ^29^, as well as differential attrition over time ^44^. With regards to the focus groups specifically, the sample size was relatively small, especially for the parent group, and it may be likely that those who agreed to attend a focus group on social media would be more willing to share their social media data. Similarly, those participants who actively participate in the study by attending focus groups may be more likely to feel positively towards the study. It is also important to recognise that a focus group methodology has drawbacks, particularly with regard to the ability to generalise the results, the ability to cover broad topics in a relatively short time-frame, and the understanding that the views of participants are socially constructed within the environment of the focus group itself ^45^. Our results should be interpreted with an awareness of these limitations.

## Conclusions

The focus groups have provided an insight into the views of cohort study participants on using their social media data in research. All participants agreed they would be happy to share their anonymised social media data with researchers affiliated with ALSPAC for health and social research, apart from data about their friends. Whilst there was a preference for anonymised data, most participants felt that their trust in the study would allow them to share any level of data with researchers, often motivated by the positive intention of the research. It is acknowledged that the sample that chose to attend the focus group was small and may have been biased in their willingness to agree to the hypothetical scenarios.

The engagement and willingness of the participants to discuss social media and its applications in research suggest that LPS could be a valuable source of ground-truth data, especially given the opportunity to link their social media data to other measures taken since birth. This would give researchers a valuable opportunity to learn more about who uses social media and start to study the attributes of this population.

Insights from this research can inform studies designing social media data collection strategies, particularly describing which categories of content are seen as more sensitive than others. Feedback from the participants emphasised the importance of clear information for any participants involved in the suggested research, especially with regard to the involvement of computers in accessing their data and safeguards used to protect it.

The participants’ views on which of their data they would be happy to share could be revealing if explored further, especially the distinction between accessing network data against accessing friends’ data.

This work paves the way for future work integrating social media with LPS data, which will be beneficial for both the studies and those conducting social media research.

## Data availability

### Underlying data

ALSPAC data access is through a system of managed open access. The steps below highlight how to apply for access to the data included in this research article and all other ALSPAC data. The datasets presented in this article are linked to ALSPAC project number B2934, please quote this project number during your application. The ALSPAC variable codes highlighted in the dataset descriptions can be used to specify required variables.

1. Please read the ALSPAC access policy ( https://www.bristol.ac.uk/media-library/sites/alspac/documents/researchers/data-access/ALSPAC_Access_Policy.pdf) which describes the process of accessing the data and samples in detail, and outlines the costs associated with doing so.
2. You may also find it useful to browse our fully searchable research proposals database ( https://proposals.epi.bristol.ac.uk/), which lists all research projects that have been approved since April 2011.
3. Please submit your research proposal for consideration by the ALSPAC Executive Committee. You will receive a response within 10 working days to advise you whether your proposal has been approved.

If you have any questions about accessing data, please email alspac-data@bristol.ac.uk.

The ALSPAC data management plan describes in detail the policy regarding data sharing, which is through a system of managed open access.

The study website also contains details of all the data that is available through a fully searchable data dictionary: http://www.bristol.ac.uk/alspac/researchers/data-access/data-dictionary/.

### Extended data

Open Science Framework: Online supplementary material for "Views on social media and its linkage to longitudinal data from two generations of a UK cohort study". https://doi.org/10.17605/OSF.IO/6RX2Z ^34^.

This project contains the following extended data:

- Supp_1_Slides (PPTX). Supplementary Material 1: Slides from the presentation given to participants.

### Reporting guidelines

Open Science Framework: COREQ checklist for ‘Views on social media and its linkage to longitudinal data from two generations of a UK cohort study’. https://doi.org/10.17605/OSF.IO/6RX2Z ^34^.

Extended data and the completed COREQ checklist are available under the terms of the Creative Commons Zero "No rights reserved" data waiver (CC0 1.0 Public domain dedication).
