# Supplementary material for: Views on social media and its linkage to longitudinal data from two generations of a UK cohort study
Source: Wellcome Open Res. 2020 Aug 12;5:44. Originally published 2020 Mar 12. [Version 2] doi: 10.12688/wellcomeopenres.15755.2 (PMC7459850; doi:10.12688/wellcomeopenres.15755.2)
Supplement: Peer review history

# Peer review: Reviewer response for version 2

**DOI:** 10.21956/wellcomeopenres.17818.r39934

**Published:** 2020-08-27

**Authors:** Vicki Xafis

**Affiliations:** 1 Centre for Biomedical Ethics, Yong Loo Lin School of Medicine, National University of Singapore, Singapore, Singapore

I thank the authors for carefully considering the comments and suggestions made. The responses are well considered and the amendments address the issues raised. While I understand the authors' point about generalisability, I would not express it as a limitation of qualitative research for the reason previously mentioned. I wish you the very best.

Is the work clearly and accurately presented and does it cite the current literature?

Partly

If applicable, is the statistical analysis and its interpretation appropriate?

Not applicable

Are all the source data underlying the results available to ensure full reproducibility?

Yes

Is the study design appropriate and is the work technically sound?

Yes

Are the conclusions drawn adequately supported by the results?

Partly

Are sufficient details of methods and analysis provided to allow replication by others?

Yes

Reviewer Expertise:

Some relevant areas include: Bioethics, Big Data, Data Linkage and I am a qualitative research.

I confirm that I have read this submission and believe that I have an appropriate level of expertise to confirm that it is of an acceptable scientific standard.

# Peer review: Reviewer response for version 1

**DOI:** 10.21956/wellcomeopenres.17278.r38236

**Published:** 2020-04-21

**Authors:** Vicki Xafis

**Affiliations:** 1 Centre for Biomedical Ethics, Yong Loo Lin School of Medicine, National University of Singapore, Singapore, Singapore

Thank you for a very interesting, well written paper. The data collection methods were evidently engaging and I sensed that the participants thoroughly enjoyed their participation in the research. The sheer amount of social media data that could be included in research is naturally very exciting. It is important, however, to ensure that we are listening carefully to what participants tell us. One thing that is abundantly clear in your research is that research participants develop a bond of trust with the researchers and are far more willing to engage and share data precisely because of this trust. I do not always think it is necessary to share the findings with research participants but, in this case, I would have done so because of the established relationship. This paper would certainly benefit from inclusion of quality literature on the ability to re-identify de-identified or anonymised data. I became a little concerned about the way some of the findings were reported and I think that changes need to made to accurately represent what participants were conveying to you.

1. Abstract: Background: “Participant’s long-term engagement with cohort studies, as well as the potential for their social media data to be linked to other longitudinal data, could provide novel advances but may also give participants a unique perspective on the acceptability of this growing research area.” The last part of this sentence does not entirely cohere with the rest and it is unclear what exactly is meant.
2. Introduction: In the Introduction you talk about a range of social media data. It is important to clarify from the start what kind of social media data you are specifically referring to in this study. If you want to avoid this because you asked participants what they considered to be social media, you should find a way to explain your non-commitment to specifying the kind of social media upfront.
3. “At present, longitudinal population studies (LPS) remain an untapped resource in terms of obtaining this empirical information, with social media linkage having the potential to add significant value to the data already available in such studies.” This statement is not quite clear and would benefit from reformulation. Are LPS an ‘untapped resource’ or do you mean that social media is an untapped resource which could “add significant value to the data already available in such studies”?
4. “As highlighted by Wellcome ^5^, and other major research funders ^6^, a key future direction for such studies is to conduct more data linkage using new types of data to reduce the burden on participants and maximise the benefit of the significant volume of longitudinal data already collected.” The Wellcome Trust reference does not seem to provide robust support for the statement, as far as I can tell. By ‘new types of data’ I have assumed you are referring to social media data. If so, ‘reducing the burden on participants’ is a very weak justification and seems to disregard the associated privacy intrusions.
5. There is a huge difference between there being support for data linkage and support for social media data to be linked to other records. 
6. Some consideration needs to be given to the fact that social media posts are uploaded for a different communicative purpose. When people express resignation in relation to an inability to control the use of their data, this should not be seen as permission to use it. Much of what transpires in social media is highly coercive leaving no room for users to make choices they might otherwise wish to make. 
7. Methods: “Beyond this they were not made aware of the facilitators’ specific research interests.” I am not entirely sure I understand the rationale for this. 
8. Analysis: “This methodology was influenced by ideas from grounded theory…” Could you please elaborate in what way the analysis was influenced by grounded theory when you were coding deductively? Also a minor point, the elusive apostrophe is missing from “participants views”. 
9. Page 8: “This passive social media use could be reflective of reported increases in social media being used as a vehicle for viewing content such as videos and news, along with decreases in users creating content.” This could also be a protective behaviour because they do not entirely trust social media and do not want to receive negative feedback on posts. 
10. Page 8: “In both groups there were references to using social media to research other people.” I would not use the word ‘research’ in this manner, particularly if it relates to ‘stalking’ of any kind. It is more ‘looking people up/ keeping tabs on people’. 
11. Page 8: “This could be attributed to ‘negativity-bias’, a known trend towards negative experiences in evaluative exercises, and may have been more pronounced due to the group environment ^41^.” Or it could be that they actually did have more negative views…I am not convinced that ‘negativity bias’ is the first thing that should come to mind. 
12. Page 9: “Photo data” is identifiable data, that is if it is a photo of a person. 
13. Page 10: It is important to give weight to participants’ initial reactions regarding friends’ data. The discussion, particularly about the value of the research, may have caused them to ‘conform’. Also, another elusive apostrophe missing in “When considering friends data…” 
14. Page 10: Data linkage involves highly technical methods so I would not easily relate the term to linking social media data to existing data sets. In addition, I would imagine that the data collected in the longitudinal study cannot be anonymised due to the nature of the research so linking social media data to the existing data set would render the social media data identifiable. Is this correct? 
15. Page 11: “When asked if they would share their friends’ data, participants were firm that they would not, but only minutes later, agreed they would when given a specific scenario. The participants even questioned themselves on the inconsistency of their response to this issue, and there was no overall resolution of opinions.” The way this finding has been phrased trivializes the participants’ original hesitation and response. 
16. Page 11: “It appeared that even those who did not have direct negative experiences of social media were invested in the narrative that social media can easily be detrimental, though the negativity bias associated with evaluative discussions is likely to have played a role in these views ^41^” This statement also trivializes these participants’ contributions and exposes the authors’ bias; “were invested in the narrative” has a negative connotation and your immediate assessment that negativity bias can explain away their views is concerning. 
17. Page 11: “This negative view of social media in general appeared alongside apparently contradictory specific examples of the benefits of social media, such as keeping in touch with friends or providing company to lonely older people.” There is nothing contradictory in these participants’ statements. On the contrary, they have engaged in perfectly expected behaviours. They recognise the benefits of social media and the negative aspects and have weighed up the two and made the assessment that they were willing to risk the negatives for the benefits social media offers. 
18. Page 12: “Interestingly, this contradicts findings by Wellcome ^25^ on the public’s views of general data linkage, where younger people were more likely to agree to share their data, and older generations were less likely. A broader sample of participants would be helpful in order to thoroughly investigate the nuance in the reasoning of both groups.” This often relates to the kind of data being shared. There is quite a lot of literature available on this. 
19. Page 12: The aim of qualitative research is not necessarily to “generalise the results”.

Is the work clearly and accurately presented and does it cite the current literature?

Partly

If applicable, is the statistical analysis and its interpretation appropriate?

Not applicable

Are all the source data underlying the results available to ensure full reproducibility?

Yes

Is the study design appropriate and is the work technically sound?

Yes

Are the conclusions drawn adequately supported by the results?

Partly

Are sufficient details of methods and analysis provided to allow replication by others?

Yes

Reviewer Expertise:

Some relevant areas include: Bioethics, Big Data, Data Linkage and I am a qualitative research.

I confirm that I have read this submission and believe that I have an appropriate level of expertise to confirm that it is of an acceptable scientific standard, however I have significant reservations, as outlined above.

# Response

**Published:** 2020-08-03

**Authors:** Nina Di Cara

**Affiliations:** University of Bristol, UK

Thank you for the constructive and thorough comments on the manuscript that have helped us to improve the clarity throughout, and particularly in reference to the reporting of participant's views. Please find our full response to your review below:

**1. **Abstract: Background: “Participant’s long-term engagement with cohort studies, as well as the potential for their social media data to be linked to other longitudinal data, could provide novel advances but may also give participants a unique perspective on the acceptability of this growing research area.” The last part of this sentence does not entirely cohere with the rest and it is unclear what exactly is meant.

**Response**: In order to clarify this sentence we have removed the middle section regarding ‘novel advances’, in order to focus it on the participant experience. 

*“Participant’s long-term engagement with cohort studies, as well as the potential for their social media data to be linked to other longitudinal data, may give them a unique perspective on the acceptability of this growing research area.”*

**​​​​​​2.** Introduction: In the Introduction you talk about a range of social media data. It is important to clarify from the start what kind of social media data you are specifically referring to in this study. If you want to avoid this because you asked participants what they considered to be social media, you should find a way to explain your non-commitment to specifying the kind of social media upfront.

**Response: **Thank you for noticing this, we have added the following sentence to the end of the first paragraph:

*“* *We note that* *the definition of the term ‘social media’ is left to be explored and defined by the participants within this study”*

**3.** “At present, longitudinal population studies (LPS) remain an untapped resource in terms of obtaining this empirical information, with social media linkage having the potential to add significant value to the data already available in such studies.” This statement is not quite clear and would benefit from reformulation. Are LPS an ‘untapped resource’ or do you mean that social media is an untapped resource which could “add significant value to the data already available in such studies”?

**Response: **To provide more clarity to this sentence we have rephrased it as follows:

*“At present, longitudinal population studies (LPS) remain an untapped resource in terms of obtaining this empirical information. Conducting social media data linkage in this way also has the potential reciprocal benefit of adding significant value to the data already available in the LPS.”*

**4. **“As highlighted by Wellcome5, and other major research funders6, a key future direction for such studies is to conduct more data linkage using new types of data to reduce the burden on participants and maximise the benefit of the significant volume of longitudinal data already collected.” The Wellcome Trust reference does not seem to provide robust support for the statement, as far as I can tell. By ‘new types of data’ I have assumed you are referring to social media data. If so, ‘reducing the burden on participants’ is a very weak justification and seems to disregard the associated privacy intrusions.

**Response: **Thank you, we agree that the links to the reports mentioned in this passage could be much clearer. In the Wellcome report we were specifically referring to section 3.2 on page 16-17 which outlines potential benefits of data linkage, and the specific references to linking social media data are in the MRC report (4.2.2, page 12, 4.2 page 16). While the MRC consider the idea of linking social media they also recommend that this is explored on a case-by-case basis, and that the quality and added value of such data is investigated.

We have rephrased the highlighted text in the hope that it is clearer, and the next paragraph then goes on to discuss the need for exploration of the ethics and social licence for this kind of data collection.

*“As highlighted by Wellcome ^5^, and the Medical Research Council (MRC) ^6^, a key future direction for such studies is to conduct more data linkage. Data linkage within existing and prospective datasets has the potential to reduce the burden on participants and maximise the benefit of research data collected ^5^, whilst using new types of data that allow for remote data capture, such as social media, is hypothesised by the MRC as a method that could address a lack of engagement and offer cost-effective modes of data collection ^6^.”*

**5.** There is a huge difference between there being support for data linkage and support for social media data to be linked to other records.

**Response: ** We agree with this statement, and it is for this reason that we note that acceptability of data linkage is ultimately complex and conditional on the situation. For clarity we have added:

*“Existing research in the field of record linkage, outside of social media linkage, has found that….”*

**6.** ****Some consideration needs to be given to the fact that social media posts are uploaded for a different communicative purpose. When people express resignation in relation to an inability to control the use of their data, this should not be seen as permission to use it. Much of what transpires in social media is highly coercive leaving no room for users to make choices they might otherwise wish to make.

**Response: **We agree that explicit consent is important when using social media data, perhaps particularly so in the case of an on-going cohort study where data collection that risks the trust between participant and researcher can have knock-on consequences for follow-up data collection. We note the particular challenge of trust and consent when working with LPS in the following sentence on the fourth paragraph of the introduction:

*“This may mean their agreement to share their data and link it to existing data is more likely. However, it is particularly important in these studies to maintain the trust that has been built with participants by co-creating an understanding of what is acceptable with regards to their information, especially since LPS participants may have specific concerns about the linking of their social media activities to the large volumes of diverse sets of data already held about them by the study.”*

**7.** Methods: “Beyond this they were not made aware of the facilitators’ specific research interests.” I am not entirely sure I understand the rationale for this.

**Response: **The focus group did not touch on other aspects of the research being done by the facilitators, and this was reported as advised in point 8 of the CORE-Q criteria for reporting qualitative research, “Interviewer characteristics” ( https://academic.oup.com/intqhc/article/19/6/349/1791966). We have also added an explanation in the text that this is being reported in line with the CORE-Q criteria:

*“* *Beyond this they were not made aware of the facilitators’ specific research interests, which we are reporting in line with the CORE-Q criteria for qualitative research ^46^.* *”*

**8.** Analysis: “This methodology was influenced by ideas from grounded theory…” Could you please elaborate in what way the analysis was influenced by grounded theory when you were coding deductively? Also a minor point, the elusive apostrophe is missing from “participants views”.

**Response: **Thank you for noting this - it was not explained as clearly as it could be. To clarify, the focus group data was split into the two parts, one of these parts (Part 3) had a clear existing structure which we coded the data into. However, Part 1 was more open and exploratory in terms of the questions being asked, and so data within this section was coded inductively into themes, in a manner influenced by grounded theory. We have clarified this paragraph below.

*“…organising it into themes.* *Due to the semi-directed nature of the focus group, and relatively small volume of data, the data were first coded deductively ^35^* *into the existing structure given by Part 1 and Part 3 of the focus groups above, rather than inductively deriving broad themes. Within the sub-theme ‘What are social media used for?’, participant responses were inductively coded and summarised into themes. As such the results are presented as narrative summaries of participants of the two parts, with any identified sub-themes as relevant.”*

**9**. Page 8: “This passive social media use could be reflective of reported increases in social media being used as a vehicle for viewing content such as videos and news, along with decreases in users creating content.” This could also be a protective behaviour because they do not entirely trust social media and do not want to receive negative feedback on posts.

**Response: **We agree that this could be a reason for users creating less content and have added this point to the sentence highlighted.  

*“* *This passive social media use could be reflective of reported increases in social media being used as a vehicle for viewing content such as videos and news, along with decreases in users creating content ^39^* *which may be to avoid negative feedback on their own user-generated content”*

**10**. Page 8: “In both groups there were references to using social media to research other people.” I would not use the word ‘research’ in this manner, particularly if it relates to ‘stalking’ of any kind. It is more ‘looking people up/ keeping tabs on people’.

**Response: **We welcome this suggestion with regards to the wording. The sentence has been amended to read:

*“In both groups there were references to using social media to find information about, or posts from, other people.”*

**11**. Page 8: “This could be attributed to ‘negativity-bias’, a known trend towards negative experiences in evaluative exercises, and may have been more pronounced due to the group environment41.” Or it could be that they actually did have more negative views…I am not convinced that ‘negativity bias’ is the first thing that should come to mind.

**Response: **Thank you for this point - we have removed the sentence referring to negativity-bias so that the participant’s views are presented in a more objective way.

**12.** Page 9: “Photo data” is identifiable data, that is if it is a photo of a person.

**Response: **Anonymity with reference to photo data refers to options such as the hues and colours used in the data, or automated descriptions of a photo such as ‘contains two people’.  We have clarified this in the text:

*“Photo data created more discussion and the younger participants generally felt they would require more anonymity with photo and location data. Anonymity of photo data in this sense referred to distilling a photo down to numeric data, such as the hues and colours used in an image, or to a description of the image.”*

**13.** Page 10: It is important to give weight to participants’ initial reactions regarding friends’ data. The discussion, particularly about the value of the research, may have caused them to ‘conform’. Also, another elusive apostrophe missing in “When considering friends data…”

**Response: **In response to this feedback we have reworded the paragraph in question (including rectifying the grammatical error):

*“* *When considering friends’ data, this did not differ across platforms, and participants were mostly consistent that they would not provide consent for this. This was with the exception of the sub-group who expressed more willingness to consent to their friend’s data being collected from Twitter, as a public platform, than Facebook. This subgroup ultimately stated that they would consent to this data being collected from both platforms if it was being used for ‘good’, though their initial reaction was that this would not be acceptable to them.”*

**14.** Page 10: Data linkage involves highly technical methods so I would not easily relate the term to linking social media data to existing data sets. In addition, I would imagine that the data collected in the longitudinal study cannot be anonymised due to the nature of the research so linking social media data to the existing data set would render the social media data identifiable. Is this correct?

**Response: **We have used the definition of data linkage provided by Wellcome in their report ( https://wellcome.ac.uk/sites/default/files/enabling-data-linkage-to-maximise-value-of-public-health-research-data-phrdf-mar15.pdf

), which defines data linkage as ‘bringing together two or more sources of information which relate to the same individual, event, institution or place’. We believe this is an accurate definition of data linkage that also applies to social media linkage, though recognise that some instances can be very complex.

In ALSPAC access to participant data is controlled by a central cohort study team, and only those data relevant to each individual research project is approved for use by a researcher. The central safe data haven for the study does contain all the linked and identifiable information about each participant in a protected environment that is not accessible to researchers.

**15.** Page 11: “When asked if they would share their friends’ data, participants were firm that they would not, but only minutes later, agreed they would when given a specific scenario. The participants even questioned themselves on the inconsistency of their response to this issue, and there was no overall resolution of opinions.” The way this finding has been phrased trivializes the participants’ original hesitation and response.

**Response: **Thank you for noting this. We have made the following rewording: 

*“When first asked if they would share their friends’ data, participants were firm that they would not. However, some agreed when presented with a specific scenario. The participants noted their own difference in opinions, and despite discussion around this there was no overall resolution of opinions for any given situation.”*

**16.** Page 11: “It appeared that even those who did not have direct negative experiences of social media were invested in the narrative that social media can easily be detrimental, though the negativity bias associated with evaluative discussions is likely to have played a role in these views41” This statement also trivializes these participants’ contributions and exposes the authors’ bias; “were invested in the narrative” has a negative connotation and your immediate assessment that negativity bias can explain away their views is concerning.

**Response: **Please see response to point 17 below.

**17.** Page 11: “This negative view of social media in general appeared alongside apparently contradictory specific examples of the benefits of social media, such as keeping in touch with friends or providing company to lonely older people.” There is nothing contradictory in these participants’ statements. On the contrary, they have engaged in perfectly expected behaviours. They recognise the benefits of social media and the negative aspects and have weighed up the two and made the assessment that they were willing to risk the negatives for the benefits social media offers.

**Response: **We agree that the wording of this paragraph does not accurately represent the nuance of the reasoning of participants. As such we have revised the wording as follows:

*“The belief that social media can be detrimental extended to those participants who said that they had not directly experienced negative consequences. However, the participants’ acknowledgement of the negative side of social media was held alongside specific examples of the benefits such as keeping in touch with friends or providing company to lonely older people. This illustrates the participants’ awareness of the advantages and disadvantages of social media, and represents a considered decision to continue engaging with it.”*

**18.** Page 12: “Interestingly, this contradicts findings by Wellcome25 on the public’s views of general data linkage, where younger people were more likely to agree to share their data, and older generations were less likely. A broader sample of participants would be helpful in order to thoroughly investigate the nuance in the reasoning of both groups.” This often relates to the kind of data being shared. There is quite a lot of literature available on this.

**Response: **We have amended the wording as follows to reflect this observation:

*“A broader sample of participants would be helpful in order to thoroughly investigate the nuance in the reasoning of both groups, and how it relates to the types of data being shared.”*

**19. **Page 12: The aim of qualitative research is not necessarily to “generalise the results”.

**Response: **Whilst we agree that this is not necessarily the intention of qualitative research, we consider the lack of generalisability of qualitative research an important limitation to mention, especially for anyone considering our findings in the context of decision making (such as other cohort studies).

# Peer review: Reviewer response for version 1

**DOI:** 10.21956/wellcomeopenres.17278.r38167

**Published:** 2020-04-07

**Authors:** Erika Kispeter

**Affiliations:** 1 Institute for Employment Research, University of Warwick, Coventry, UK

This paper is an informative description of a piece of research which explored how two generations of participants of the ALSPAC study use and view social media and their opinions on linking social media data to the data that have been collected from them in ALSPAC. The research was well designed and the paper is very well written and easy to follow - it was a pleasure reading it.

 The paper describes the motivation for conducting the study and outlines how it contributes to the existing body of literature on public perceptions about and preferences for sharing social media data and using this type of data in data linkage for research purposes. 

 Most of the study’s findings are consistent with those discussed in the literature about data linkage and social media research. Focus group participants agreed that they would consent to ALSPAC collecting their social media data if the data were collected by ALSPAC staff and then anonymised. However, most participants felt that their trust in ALSPAC would allow them to share data of any level of anonymity, including raw data, with researchers.  Certain types of data, for example data about participants’ social media ‘friends’ were seen as more sensitive than others. The final finding of the study is that participants would consent to their social media data being linked to the data already held about them in ALSPAC – this was also motivated by the high level of trust participants had in ALSPAC.

 The research methods are described clearly and in sufficient detail. It is acknowledged that the focus group had relatively few participants and they may have been biased in their willingness to share social media data and consent to data linkage.

 A particularly interesting part of the paper focuses on the generational differences in concerns about sharing and linking social media data. The authors argue that the two groups’ different levels of technological insight are part of the explanation for the differences and that the younger generation of focus group participants are ‘digital natives’. There is also a nuanced discussion about the different, generation-specific considerations underlying the two groups’ willingness to share their social media data with researchers.

 An important limitation of the study is that focus group participants, who have been involved in ALSPAC for a long time, are characterised by a high level of awareness of the value of health research and had a high level of trust towards ALSPAC staff, which influenced their willingness to share social media data with ALSPAC. In other words, the study participants are a highly select group.

 The study is scientifically sound without further changes. However, I would make some minor suggestions which may improve an already excellent paper:

 The popular argument about 'digital natives' that is referenced in the paper has been widely challenged. In particular, Helsper and Eynon (2009) ^1^ argue that rather than age, it is the breadth of activities people carry out online that determines whether someone id a 'digital native' or not.

 The legend of Figure 1 currently reads: "This template was use by participants" - the correct form is 'used’.

Is the work clearly and accurately presented and does it cite the current literature?

Yes

If applicable, is the statistical analysis and its interpretation appropriate?

Not applicable

Are all the source data underlying the results available to ensure full reproducibility?

Yes

Is the study design appropriate and is the work technically sound?

Yes

Are the conclusions drawn adequately supported by the results?

Yes

Are sufficient details of methods and analysis provided to allow replication by others?

Yes

Reviewer Expertise:

Qualitative Research, Familiarity with the literature on public perceptions on data sharing and data linkage.

I confirm that I have read this submission and believe that I have an appropriate level of expertise to confirm that it is of an acceptable scientific standard.

## References

[1] : Digital natives: where is the evidence?. *British Educational Research Journal*.2010;36(3) : 10.1080/01411920902989227 503-520 10.1080/01411920902989227

# Response

**Published:** 2020-08-03

**Authors:** Nina Di Cara

**Affiliations:** University of Bristol, UK

Thank you for your kind comments on our manuscript. 

​​​​​​In response to your feedback regarding the reference to digital natives we have rewritten the paragraph that references this idea to more accurately reflect how the concept relates to our results:

“This could be seen as consistent with the younger group having grown up with technology available to them and having a different awareness of how it operates than their parents do. However, this view of younger generations as ‘digital natives’ [43] can be misleading; whilst age is associated with someone’s likelihood to be immersed in technology, it is not the only relevant factor [Helsper and Eynon 2009]. As such the generational differences we observed may not solely be attributed to the participant’s age.”

Thank you also for noticing the error in the legend of Figure 1, we have corrected the form from 'use' to 'used'.
